# Comparison of Surgical Outcomes between Trabeculectomy with Mitomycin C and Ahmed Valve Implantation with Mitomycin C in Eyes with Uveitic Glaucoma

**DOI:** 10.3390/jcm11051368

**Published:** 2022-03-02

**Authors:** Seung Yeop Lee, Yong Hyun Kim, Ko Eun Kim, Jaehong Ahn

**Affiliations:** 1 Department of Ophthalmology, Ajou University School of Medicine, 164 World Cup-ro, Yeongtong-gu, Suwon 16499, Korea; rommu2000@gmail.com (S.Y.L.); kim-yh87@hanmail.net (Y.H.K.); 2 Department of Ophthalmology, Asan Medical Center, University of Ulsan College of Medicine, 88 Olympic-ro 43-gil, Songpa-gu, Seoul 05505, Korea

**Keywords:** glaucoma, uveitis, trabeculectomy, Ahmed valve implantation

## Abstract

We compared 1-year outcomes of trabeculectomy with mitomycin C (MMC) and Ahmed valve implantation with MMC as a first surgical procedure in patients with uveitic glaucoma. A total 38 eyes of 38 patients undergoing trabeculectomy (*n* =16) or Ahmed valve implantation (*n* = 22) were included. Surgical success was defined as intraocular pressure (IOP) ≤21 mmHg, IOP reduction ≥20% from baseline, no secondary glaucoma surgery, and no loss of light perception. The main outcome measurements including success rate, IOP, and the number of antiglaucoma medications and complications were compared. The overall success rates were comparable between the Ahmed and trabeculectomy groups (81.3 vs. 81.8%, *p* = 0.987). The mean IOPs were similar as well (*p* = 0.084), though the number of antiglaucoma medications was significantly lower in the trabeculectomy group than in the Ahmed group (1.0 ± 1.2 vs. 2.2 ± 1.1; *p* = 0.005). A statistically significant reduction in corneal endothelial cell density was noted in the Ahmed group (*p* = 0.004). Both treatments offered reasonable IOP control and safety for eyes with uveitic glaucoma. However, significantly fewer antiglaucoma medications were used in the trabeculectomy group. Furthermore, our results suggest that cautious postoperative monitoring with regard to corneal endothelial cell density should be additionally performed after Ahmed valve implantation.

## 1. Introduction

Uveitic glaucoma is a disease caused by elevated intraocular pressure (IOP) due to increased resistance to aqueous outflow associated with the etiology of uveitis and inflammation [1,2]. The incidence of glaucoma in uveitic patients has been reported to be between 9.6% and 18.3% and as high as 46% in severe chronic uveitis [3], according to disease etiology. Medical therapy including an IOP-lowering agent is considered to be the first choice for IOP control in uveitic glaucoma. However, surgical intervention often is required, due to the detrimental effect of acute or chronic inflammation on the aqueous outflow, manifesting as aggravated outflow resistance and uncontrolled IOP elevation [2,4,5].

Ahmed valve implantation and trabeculectomy have been the primary surgical treatments for uveitic glaucoma [6]. However, the question remains as to which surgery is better performed initially. Ahmed valve implantation has been reported to be safe and effective in terms of long-term IOP control [7,8,9,10]. However, it also carries a high risk of complications including hyphema, endothelial decompensation, and tube exposure [7,8,9,11]. In the case of trabeculectomy, it has advantages in that it is revisable in cases of bleb failure; but it has also limitations due to the high probability of bleb fibrosis associated with recurrent inflammation, leading to lower success rates [12]. Previous studies have reported generally good postoperative outcomes for both of these surgical interventions [13,14,15,16]. Nonetheless, the results have been varied, and even studies directly comparing them have shown inconsistent results [17,18]. Moreover, in most of the studies, surgeries were performed by various surgeons under various conditions (e.g., mitomycin C (MMC) concentration, duration of MMC application, the definition of surgical success), rendering fair comparisons problematic [13,19,20,21].

In this regard, the purpose of this study was to compare the success rates of trabeculectomy/MMC with Ahmed valve implantation/MMC as a first surgical intervention in patients with uveitic glaucoma over a 1-year follow-up period. The surgeries were performed by a single glaucoma specialist, and, as noted above, MMC was used with both treatment modalities.

## 2. Materials and Methods

We retrospectively reviewed the medical records of patients who had undergone trabeculectomy with MMC or Ahmed valve implantation with MMC for medically uncontrolled uveitic glaucoma between June 2009 and April 2020 at Ajou University Hospital. This study was approved by the ethics committee and the Institutional Review Board of Ajou university hospital and followed the tenets of the Declaration of Helsinki.

### 2.1. Patients

Uveitic glaucoma is defined as the type of glaucoma that manifests as glaucomatous optic nerve head damage and/or visual field defect due to elevated IOP associated with increased resistance to aqueous outflow following inflammation [22]. Uveitis was diagnosed based on clinical examination, and its type was classified according to the criteria of the International Uveitis Study group [23]. The grading of the anterior chamber cell was performed based on the standardized uveitis nomenclature scheme as follows: a complete absence of cells with 0, barely detectable cells with 1+, moderate severity of anterior chamber cells with 2+, marked severity with 3+, and intense severity with 4+ [23].

All of the patients received complete ophthalmic examinations including slit-lamp examination, IOP measurement with Goldmann applanation tonometry, gonioscopy, and dilated fundus examination. They also underwent cornea specular microscopy (SP-2000P; Topcon Corp., Tokyo, Japan), disc and fundus photography (Visucam 224; Carl Zeiss Meditec Inc., Dublin, CA, USA), spectral-domain optical coherence tomography (Cirrus; Carl Zeiss Meditec Inc.), and 24-2 Swedish interactive threshold algorithm standard automated perimetry (Humphrey Visual Field Analyzer 3; Carl Zeiss Meditec Inc.). The inclusion criteria were as follows: (1) uveitic glaucoma having medically uncontrolled IOP of > 21 mmHg despite administration of maximally tolerated IOP-lowering medication on two consecutive visits, (2) vision better than light perception, (3) no previous history of glaucoma surgery, and (4) a minimum follow-up period of 1 year postoperatively. The exclusion criteria included ocular comorbidities—namely, optic neuropathy other than uveitic glaucoma, macular disease, and retinopathy that may interfere with data results.

### 2.2. Outcome Measures

The main postoperative outcome measurements included IOP, the number of antiglaucoma medications, best-corrected vision acuity (BCVA), and surgical complications. Follow-up visits were scheduled at 1 day, 1 week, 2 weeks, 1 month, 3 months, 6 months, 9 months, and 1 year after surgery. At each visit, visual acuity (VA), IOP, microscopic findings, complications, and the number of antiglaucoma medications were assessed. Data on BCVA, IOP, and the number of antiglaucoma medications were collected at baseline visits prior to surgery and at 6 and 12 months after surgery in order to better compare surgical success. Corneal endothelial cell densities at baseline visits and postoperatively at 6 and 12 months were recorded for comparison of surgical complications related to the cornea. Decimal VA was converted to the minimum angle of resolution (logMAR). Any fixed-dose combination drugs were considered to be two separate drugs.

Surgical failure was defined as follows: (1) IOP > 21 mmHg or reduced by less than 20% from the baseline on 2 consecutive follow-up visits after 3 months; (2) IOP of 5 mmHg or less on 2 consecutive follow-up visits after 3 months; (3) reoperation due to inadequately controlled IOP; (4) loss of light perception.

Overall success was considered to be represented by patients for whom none of the above criteria was applicable. Complete success was represented by those who had not failed and were not administered antiglaucoma medication. Qualified success was represented by those who had not failed but had received antiglaucoma medication. Reoperation was defined as additional glaucoma surgery necessitated by inadequate IOP control. Interventions such as laser suture lysis or bleb needling were considered to be normal postoperative care and therefore was not recorded as either reoperation or failure. Conjunctival suture to prevent ocular hypotony due to wound leakage likewise was not considered to be a case of reoperation, but rather, a complication. Acute hypotony was defined as IOP < 6 mmHg within the first month of follow-up, and wound leak was determined by Seidal test positivity within the first month of follow-up. The hypertensive phase was defined as an IOP of > 21 mmHg within 6 months postoperatively. Cataract progression was defined as a loss of visual acuity attributable to cataracts or eyes that underwent cataract surgery within 1 year of follow-up.

### 2.3. Surgical Procedures

Surgery was performed by an experienced glaucoma specialist (J.A.) using standard documented techniques. Ahmed valve implantation was performed in the superotemporal quadrant. There, the conjunctiva was opened and the bare sclera was dissected. A sponge soaked with 0.04% MMC was applied for 5 min to the bare sclera, followed by irrigation with balanced salt solution (BSS). The edge of the plate of the Ahmed valve (model FP-7; New World Medical Inc., Rancho Cucamonga, CA, USA) was secured to the sclera 8 to 9 mm posterior to the limbus with two 7–0 prolene sutures, after priming the Ahmed valve with BSS and making a partial ligation of the tube with 8–0 vicryl 6 to 8 mm posteriorly [24]. The tube was trimmed bevel-up and inserted into the anterior chamber through a needle incision. In two patients, the tube was inserted into the sulcus to protect the corneal endothelium. It was covered with a 4 × 4 mm human-donor scleral patch that was fixed with 10–0 nylon. Tenon’s capsule and the conjunctiva were closed over the Ahmed valve using 10–0 nylon.

Trabeculectomy was performed superiorly following limbal conjunctival peritomy (fornix based). A 3 × 3 mm scleral flap was created, under which a sponge soaked with 0.04% MMC was then applied for 3 min. After irrigation using BSS, a block of tissue anterior to the scleral spur was removed by Kelly punch, and then peripheral iridectomy was performed. The scleral flap was closed with two releasable 10–0 nylon sutures to adjust for adequate filtration. The conjunctiva was closed using 10–0 nylon sutures.

Postoperatively, all patients, regardless of which surgical procedure they had undergone, were treated with the following topical medications: 0.5% levofloxacin hydrate (Cravit, Santen, Osaka, Japan) 4 times per day, 1% prednisolone acetate (Pred Forte, Allergan, Irvine, CA, USA) 4 times per day, and bromfenac sodium hydrate (Bronuck Oph Soln, Taejoon, Seoul, Korea) twice daily, all for six months, and all of which subsequently were gradually tapered by the glaucoma specialist.

### 2.4. Statistical Analysis

For comparison of baseline characteristics between the two groups, Student’s *t*-test and Mann–Whitney U test were applied for the numerical variables, and the chi-squared and Fisher’s exact tests were run for the categorical data. The IOP, VA, corneal endothelial cell density, and antiglaucoma medication changes before and after surgery were evaluated within each group using the Wilcoxon signed-rank test and paired *t*-test. For evaluation of serial IOP changes during the preoperative and follow-up periods, the repeated-measure one-way analysis of variance (ANOVA) test was used. We used Kaplan–Meier survival analysis to calculate the cumulative rate of surgical success in each group and the log-rank test for the inter-group comparison. The univariate and multivariate Cox proportional hazard models were used to estimate the possible risk factors associated with surgical failure. After univariate analysis, variables with *p* < 0.1 were entered into the multivariate analysis by Cox regression analysis. All numerical variables are presented herein as mean ± standard deviations unless otherwise noted. *p* < 0.05 was considered to represent statistical significance. All of the statistical analyses were performed with SPSS software (IBM SPSS Statistics for Windows version 25.0, IBM Corp., Armonk, NY, USA).

## 3. Results

### 3.1. Baseline Characteristics

A total of 38 eyes of 38 patients (16 Ahmed valve implantation with MMC, 22 trabeculectomy with MMC) were evaluated. Their baseline characteristics are provided in Table 1. No significant difference was found between the two groups regarding age, gender, preoperative IOP, preoperative inflammation severity, or preoperative corneal endothelial cell density. The Ahmed group showed significantly higher values for preoperative VA and the number of eyes with previous cataract history. Idiopathic uveitis was the most common uveitis type, comprising 75.0% of the Ahmed group and 63.6% of the trabeculectomy group; there were no significant uveitis-type differences between the groups (*p* = 0.46).

### 3.2. Surgical Success

**Figure 1:**
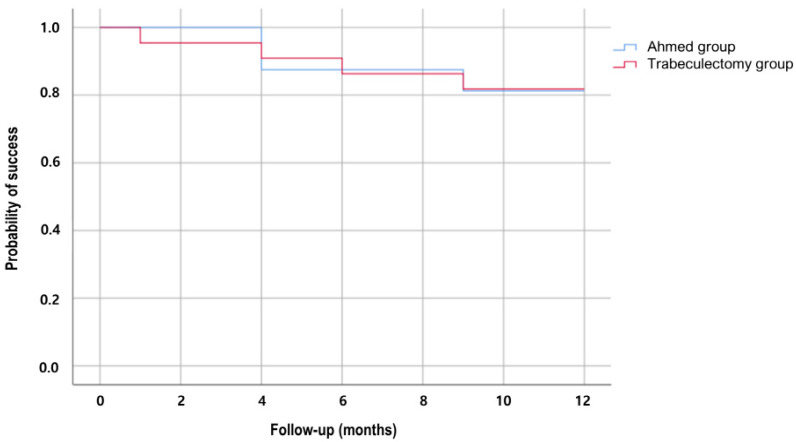
Kaplan–Meier plot showing the cumulative probability of success after 1 year in the Ahmed group versus trabeculectomy group. The success rates at 1-year follow-up were 81.3% in the Ahmed group and 81.8% in the trabeculectomy group (Log-rank test, *p* = 0.987).

The cumulative probabilities of overall success were 87.5% in the Ahmed group and 86.4% in the trabeculectomy group at 6 months (*p* = 0.905), and 81.3% in the Ahmed group and 81.8% in the trabeculectomy group at 1 year (*p* = 0.987; Figure 1). The complete success rate was significantly higher in the trabeculectomy group at both 6 months (81.8 vs. 18.8%, *p* < 0.001) and 1 year (50.0 vs. 12.5%, *p* = 0.016; Table 2). By contrast, the qualified success rate was significantly higher in the Ahmed group throughout the follow-up period (all *p* < 0.05).

At the one-year postoperative follow-up, three eyes (18.8%) in the Ahmed group and four eyes (18.2%) in the trabeculectomy group showed surgical failure (Table 2). The mean time to failure was 5.7 ± 2.9 months in the Ahmed group and 5.0 ± 3.4 months in the trabeculectomy group (*p* = 0.857). Inadequate IOP reduction occurred in two eyes (12.5%) in the Ahmed group and in one eye (4.5%) in the trabeculectomy group. One eye (6.3%) from the Ahmed group and three eyes (13.6%) from the trabeculectomy group underwent reoperation. None of the patients from either group showed persistent hypotony or loss of light perception.

### 3.3. Intraocular Pressure and Number of Antiglaucoma Medications

**Figure 2:**
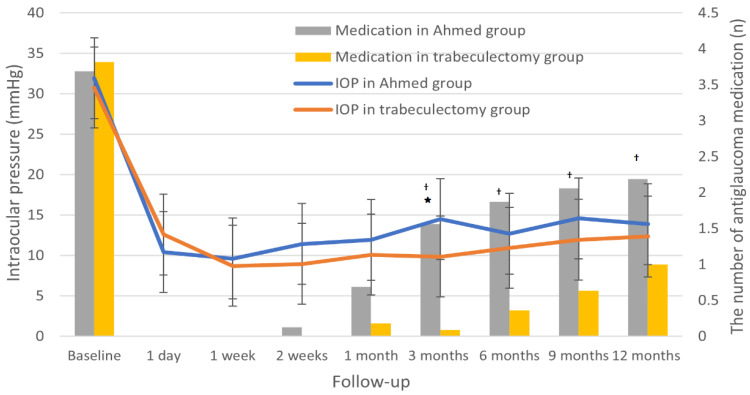
Intraocular pressure (IOP) at baseline and during postoperative follow-up period in the Ahmed group versus trabeculectomy group. Mean IOPs during follow-up period were comparable between Ahmed and trabeculectomy groups (*p* > 0.05), except at 3 months postoperation, when the Ahmed group showed significantly higher mean IOP than trabeculectomy group (* *p* = 0.001). The number of antiglaucoma medications was significantly higher in the Ahmed group at postoperative follow-up of 3, 6, 9 (*p* < 0.001), and 12 months (^†^ *p* = 0.007).

Figure 2 shows the baseline and follow-up IOP measurements for the Ahmed and trabeculectomy groups. By 1-year postoperation, mean IOP had decreased significantly from 31.9 ± 9.9 to 13.9 ± 3.2 mmHg in the Ahmed group (*p* < 0.001) and from 30.8 ± 7.9 to 12.4 ± 5.1 mmHg in the trabeculectomy group (*p* < 0.001). Mean postoperative IOP in the Ahmed group was slightly higher than that in the trabeculectomy group, though the difference generally was statistically insignificant through the follow-up period (*p* > 0.05), except at 3 months postoperation (*p* = 0.001). The hypertensive phase occurred in 12.5% in the Ahmed group and 4.5% in the trabeculectomy group (*p* = 0.314).

A significant reduction in the number of postoperative antiglaucoma medications was found in both groups at the 1-year follow-up (Figure 2; Table 3). The number of antiglaucoma medications significantly decreased, from 3.7 ± 0.6 to 2.2 ± 1.1 in the Ahmed group (*p* = 0.001) and from 3.8 ± 0.4 to 1.0 ± 1.2 in the trabeculectomy group (*p* < 0.001). However, the intergroup comparison during the follow-up period showed a significantly lower number of antiglaucoma medications in the trabeculectomy group at 3, 6, 9, and 12 months postoperation (all *p* < 0.05), except at 1-month postoperation (*p* = 0.145).

### 3.4. Visual Acuity, Severity of Inflammation, and Corneal Endothelial Cell Density

Neither of the groups showed any significant change in VA at 1-year postoperative follow-up, compared with the baseline (all *p* > 0.05). Both groups showed decreased inflammation and a similar inflammatory status at 1-year postoperation. However, a significant reduction in inflammation was seen only in the Ahmed group at 1-year postoperative follow-up, compared with the baseline (*p* = 0.019). A statistically significant reduction in corneal endothelial cell density was noted in the Ahmed group at 1-year follow-up, in contrast to the trabeculectomy group (*p* = 0.004).

### 3.5. Surgical Complications

Postoperative complications were found in 5 eyes (31.3%) and 11 eyes (50.0%) in the Ahmed and trabeculectomy groups, respectively, showing no significant difference between them (*p* = 0.682; Table 4). The most common complication in the Ahmed group was acute hypotony (12.5%), which necessitated anterior chamber Healon injection. In the trabeculectomy group, cataract progression (27.3%) was the most common complication. Corneal decompensation due to corneal-tube touch was observed in one eye (6.3%) from the Ahmed group, which later required penetrating keratoplasty. Bleb needling was not considered to reflect a postoperative complication in our study, but the trabeculectomy group showed a higher but statistically insignificant rate (50.0 vs. 18.8%, *p* = 0.09).

### 3.6. Risk Factors Associated with Surgical Failure in Uveitic Glaucoma

The surgery type (Ahmed valve implantation versus trabeculectomy), gender, age, preoperative IOP, preoperative VA, preoperative corneal endothelial cell density, preoperative antiglaucoma medication, preoperative inflammation, and previous history of cataract operation were entered into a Cox proportional hazard regression analysis. After univariate and multivariate analyses, previous history of cataract operation was the only significant risk factor associated with surgical failure (odds ratio = 0.109, 95% confidence interval = 0.013–0.908, *p* = 0.040). The type of surgery showed no significant association with surgical failure.

## 4. Discussion

Our study showed comparable success rates between Ahmed valve implantation with MMC and trabeculectomy with MMC in patients with uveitic glaucoma throughout the 1-year follow-up period. The two surgical methods also showed similar postoperative outcomes in terms of IOP, VA, and postoperative complications during the follow-up period. However, trabeculectomy showed a significantly higher complete success rate and a significantly reduced number of postoperative antiglaucoma medications, compared with Ahmed valve implantation with the help of bleb needling.

We found that trabeculectomy with MMC and Ahmed valve implantation with MMC had similar overall success rates at postoperative 6 months and 1 year. These results are in line with those of two previous comparative analysis studies [18,25]. However, a study by Bettis et al. [17], who compared 16 cases of trabeculectomy with 24 cases of Ahmed valve implantation (similarly to our study), showed significantly different surgical success rates at 1 year—namely, 100% in the Ahmed group but only 66.7% in the trabeculectomy group. Their lower success rate for the trabeculectomy group was probably attributable to their inclusion of second-trabeculectomy cases carrying a higher risk of failure and their consideration of bleb needling as a surgical failure.

Despite similar postoperative success rates between the trabeculectomy and Ahmed groups, the complete success rate, based on stricter IOP criteria, was significantly higher in the trabeculectomy group, indicating potentially different inter-treatment advantages for the treatment of uveitic glaucoma. We found that trabeculectomy had a significant benefit over Ahmed valve implantation—namely, its lower postoperative IOP values, as achieved with significantly fewer antiglaucoma medications. Audrey et al. [18] reported similar results to ours: significantly worse IOP control and a higher number of antiglaucoma medications in the Ahmed group, compared with the trabeculectomy and Baerveldt groups. Our Ahmed group had a higher incidence of hypertensive phase, which lasted from 3 weeks to several months after surgery. This could have contributed to their higher postoperative IOP and reuse of antiglaucoma medications throughout the follow-up period. As for bleb needling, it was not regarded as a surgical failure in our study, though it may be a burden to some patients and clinicians. Thus, longer-term follow-up may be required in order to supplement our results to show that bleb needling could effectively revive bleb function and maintain adequate IOP without antiglaucoma medication even in patients with uveitic glaucoma.

For patients with uveitic glaucoma, some of the most commonly reported complications have been hypotony, corneal edema, and hyphema for Ahmed valve implantation [9], and aqueous leakage, macular edema, and cataract progression for trabeculectomy [13]. Acute hypotony was the most common complication found in our Ahmed group, as is consistent with previous studies [8,21]. Corneal decompensation is another serious and common complication after Ahmed valve implantation. Although the incidence of corneal complications leading to severe visual change was relatively low in our series, compared with previous studies [9,10], we found a significant reduction in corneal endothelial cell density, especially in the Ahmed group at 1-year follow-up. However, in the present study, this may also be associated with the relatively lower baseline corneal endothelial cell count (regardless of significance) and the higher portion of patients having previous cataract surgery in the Ahmed group. These facts indicate that cautious postoperative follow-up is required for patients who have undergone Ahmed valve implantation as well as for those with a low baseline corneal endothelial cell count and a history of previous intraocular surgery. Cataract progression is one of the most common late post-trabeculectomy complications in patients with uveitic glaucoma [7,26,27], as noted also in our study. This is associated mainly with chronic inflammation and the use of topical corticosteroids. However, in our study, the significantly lower proportion of pseudophakic eyes in the trabeculectomy group, compared with the Ahmed group (22.7 vs. 81.2%), could have been another attributable factor.

Recurrence of inflammation may be crucial in patients with uveitic glaucoma, as it has been reported to be the major cause of its lower surgical success rates compared with primary open-angle glaucoma [28]. In another study, relatively high inflammation recurrence rates were reported, specifically 17.6% for trabeculectomy/MMC and 37.5% for Ahmed valve implantation, which significantly lowered the overall surgical success rates [17]. In the present study, most of our patients had relatively controlled uveitis preoperatively, and there was no case of recurrent inflammation during the follow-up period. This is likely the reason for the relatively low surgical failure and low complication rates in our study, compared with those in previous studies. These suggest that thorough control of preoperative and postoperative inflammation would be helpful for better maintenance of IOP and surgical success in uveitic glaucoma.

Previous studies have reported variable risk factors associated with surgical failure in uveitic glaucoma after trabeculectomy, including immediate postoperative IOP spike, early laser suture lysis [26], male sex [19], and postoperative exacerbation of inflammation [29]. Our results indicated that previous history of cataract operation was the only protective factor against surgical failure in patients with uveitic glaucoma. The reason for such association is not yet proven, and in fact, the results as reported in the relevant prior studies have been contradictory. For example, one study found no association between previous cataract surgery and trabeculectomy [29], whereas another study reported that previous cataract surgery had a negative effect on both trabeculectomy and Ahmed valve implantation, albeit with only marginal significance [17]. We performed an additional comparative analysis between pseudophakic (*n* = 17) and phakic (*n* = 21) eyes, but no difference in their baseline characteristics was found. Although we tried to perform risk factor analyses separately for trabeculectomy and Ahmed valve implantation, it was impossible due to the small number of patients. Further investigation with a larger number of patients will be needed to confirm the possible association between pseudophakic eyes and glaucoma surgery in uveitic glaucoma.

There are several limitations to this study. First, due to its retrospective nature and relatively small sample size, the results might not be completely representative of the general population. This notwithstanding, the study has advantages in that the surgeries were all performed by the same surgeon using the same technique and the same concentration of antiproliferative agent (MMC), for both groups. Second, the 1-year postoperative follow-up period might not be sufficient for comparison of the two surgical methods’ outcomes. Although the follow-up period might indeed seem short, our results nonetheless are suggestive of what precautions to take during the first postoperative year in order to maintain and increase the surgical success rate for uveitic glaucoma. Third, and finally, randomization was not performed when dividing the patients into the trabeculectomy and Ahmed valve groups, which may be a potential source of selection bias in the study. Nonetheless, the patients satisfying the inclusion criteria were consecutively included in the study, and the Ahmed valve implantation and trabeculectomy were performed evenly during the time period. For a more accurate comparison of the two surgical methods, a prospective randomized controlled study will be necessary.

## 5. Conclusions

In conclusion, trabeculectomy/MMC and Ahmed valve implantation/MMC in eyes with uveitic glaucoma had similar overall success rates throughout the 1-year follow-up period. The trabeculectomy showed a significantly higher complete success rate and a lower number of antiglaucoma medications after 1 year. Moreover, our results imply that cautious postoperative monitoring of corneal endothelial cell density is crucial after Ahmed valve implantation. Furthermore, a prospective, randomized study with a larger population is necessary to more accurately compare the utility of these surgical methods for eyes with uveitic glaucoma.

## Figures and Tables

**Table 1:** Baseline characteristics of Ahmed group versus trabeculectomy group.

Characteristics	Ahmed Group (*n* = 16)	Trabeculectomy Group (*n* = 22)	*p*-Value
Age, yrs	51.1 ± 17.2	47.2 ± 13.9	0.46
Male, *n* (%)	14 (87.5)	17 (77.3)	0.68
Right eye, *n* (%)	11 (68.8)	9 (40.9)	0.09
Preoperative IOP, mmHg	31.9 ± 9.9	30.8 ± 7.9.	0.69
Preoperative VA, logMAR	0.60 ± 0.57	0.28 ± 0.30	0.03
Preoperative inflammatory anterior chamber cell, *n*	1.2 ± 1.2	0.8 ± 0.9	0.28
Preoperative antiglaucoma medication, *n*	3.7 ± 0.6	3.8 ± 0.4	0.69
Preoperative corneal endothelial cell density, mm^2^	2036.9 ± 856.1	2263.0 ± 499.9	0.31
Prior cataract surgery, *n* (%)			<0.001
No	3 (18.8)	17 (77.3)	
Yes	13 (81.3)	5 (22.7)	
Duration of uveitis before surgery, m	5.2 ± 3.4	5.4 ± 3.2	0.848
Uveitis etiology, *n* (%)			0.46
Idiopathic	12 (75.0)	14 (63.6)	
Herpes simplex virus	1 (6.3)	3 (13.6)	
Cytomegalovirus	0	2 (9.0)	
Posner Schlossman syndrome	2 (12.5)	1 (4.5)	
Ocular tuberculosis	1 (6.3)	0	
Sarcoidosis	0	1 (4.5)	
Behcet’s disease	0	1 (4.5)	

IOP: intraocular pressure; VA: visual acuity; logMAR: logarithm of the minimum angle of resolution.

**Table 2:** Comparison of postoperative complete success rate, qualified success rate, and failure rate, between the Ahmed and trabeculectomy groups.

Rates	6 Months			12 Months		
	Ahmed Group (*n* = 16)	Trabeculectomy Group (*n* = 22)	*p*-Value	Ahmed Group (*n* = 16)	Trabeculectomy Group (*n* = 22)	*p*-Value
Complete success	3 (18.8)	18 (81.8)	<0.001	2 (12.5)	11 (50.0)	0.016
Qualified success	11 (68.8)	1 (4.5)	<0.001	11 (68.8)	7 (31.8)	0.024
Failure	2 (12.5)	3 (13.6)	0.098	3 (18.8)	4 (18.2)	0.094

Data are presented as number (%).

**Table 3:** Preoperative and postoperative change in visual acuity, anterior chamber inflammation, number of antiglaucoma medication, corneal endothelial cell density in the Ahmed group and trabeculectomy group.

Variables	Ahmed Group (*n* = 16)				Trabeculectomy Group (*n* = 22)				*p*-Value ^†^
	Baseline	6 Months	1 Year	*p*-Value *	Baseline	6 Months	1 Year	*p*-Value *	
VA, logMAR	0.60 ± 0.57	0.67 ± 0.95	0.62 ± 0.83	0.78	0.28 ± 0.30	0.21 ± 0.27	0.23 ± 0.30	0.567	0.23
Anterior chamber cell, *n*	1.2 ± 1.2	0.8 ± 0.7	0.4 ± 0.5	0.019	0.8 ± 0.9	0.9 ± 1.1	0.6 ± 0.7	0.518	0.53
Antiglaucoma medication, *n*	3.7 ± 0.6	1.8 ± 1.1	2.2 ±1.1	0.001	3.8 ± 0.4	0.4 ± 0.8	1.0 ± 1.2	<0.001	0.007
Corneal endothelial cell density, mm^2^	2036.9 ± 856.1	1738.2 ± 471.9	1713.6 ± 541.6	0.004	2263.0 ± 499.9	2380.9 ± 439.7	2297.4 ± 616.0	0.881	0.012

* Comparison between data at baseline and postoperative 1 year. ^†^ Comparison between the two groups at postoperative 1 year.

**Table 4:** Comparison of surgical complications between Ahmed and trabeculectomy groups.

	Ahmed Group (*n* = 16)	Trabeculectomy Group (*n* = 22)	*p*-Value
No, *n* (%)	11 (68.8)	11 (50.0)	0.682
Yes, *n* (%)	5 (31.3)	11 (50.0)	
Acute hypotony	2 (12.5)	1 (4.5)	
Cystoid macular edema	0	1 (4.5)	
Wound leakage	1 (6.3)	3 (13.6)	
Cataract progression	1 (6.3)	6 (27.3)	
Corneal decompensation	1 (6.3)	0	

## Data Availability

The data presented in this study are available from the corresponding author upon reasonable request.
